# Exploring the Properties and Application Potential of β‐Glucan in Skin Care

**DOI:** 10.1002/fsn3.70212

**Published:** 2025-04-25

**Authors:** Xiaoyue Feng, Jianli Shang, Yuhui Wang, Yong Chen, Youting Liu

**Affiliations:** ^1^ R&D Department Beijing UPROVEN Medical Technology co., Ltd. Beijing China; ^2^ Beijing UPROVEN Institute of Dermatology Beijing China

**Keywords:** β‐glucan, bioactivity, properties, skin applications

## Abstract

β‐glucan is a natural polysaccharide widely found in plants, fungi, bacteria, and algae. Due to its significant immunomodulatory effects, it has become an important source for functional foods and pharmaceuticals. In addition to immune regulation, β‐glucan also exhibits various bioactivities, including antioxidant, anti‐inflammatory, barrier repair, and moisturizing effects, demonstrating great potential for applications in skin care. Its biological activity is influenced by factors such as its source, molecular structure, and physicochemical properties. This review systematically explores the relationship between the properties and functions of β‐ glucan, investigates its biological mechanisms, and summarizes its clinical applications and future prospects in skin care. The aim of this paper is to provide theoretical support for the development of β‐glucan in the field of skin health and offer references for future related research and clinical practice.

## Graphical abstract

β‐glucan is a natural polysaccharide with significant immunomodulatory, antioxidant, anti‐inflammatory, and moisturizing effects, making it a promising ingredient for skin care applications. This review systematically explores its properties, biological mechanisms, and clinical applications, offering theoretical support and guidance for future research and development in skin health.

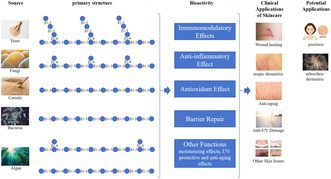

## 1 Introduction

β‐Glucan is a polysaccharide composed of D‐glucose monomers linked by β‐(1 → 3) and/or β‐(1 → 4) glycosidic bonds, widely found in plants, fungi, bacteria, and algae. As a critical component of cell walls, β‐glucan plays an essential role in maintaining cellular structure and function (Riseh et al. 2023). Due to its significant immunomodulatory effects, β‐glucan is often referred to as “the immune gold.” Research on β‐glucan dates back to the 1940s, when Pillemer and Ecker first discovered that polysaccharides in the yeast cell wall could inhibit the third component of the complement system. Subsequent studies confirmed that β‐glucan is the major constituent of these polysaccharides (Di Luzio and Riggi 1970; Pillemer and Ecker 1941).

With advancing research, β‐glucan has been shown to exhibit a wide range of biological activities, including immunomodulation, antitumor effects, cholesterol reduction, blood sugar regulation, and antimicrobial properties (Zhu et al. 2016). The U.S. Food and Drug Administration (FDA) approved β‐glucan as a safe food additive and dietary supplement in 2009 and allowed its application in the pharmaceutical field (Xiao‐xia 2012).

In recent years, the application of β‐glucan in skin care has garnered increasing attention. Studies have shown that it possesses multiple benefits, such as antioxidant, anti‐inflammatory, wound‐healing, and moisturizing effects, making it a promising candidate for the treatment of various skin conditions (Sousa et al. 2023). This review explores the relationship between the properties and functions of β‐glucan, investigates its biological mechanisms, and summarizes the latest research on its applications in skin care. Existing studies suggest that β‐glucan demonstrates positive effects in the treatment of skin issues such as wound healing, atopic dermatitis, photoaging, and ultraviolet (UV) damage. We believe this is the first systematic review summarizing the clinical applications of β‐glucan in dermatology, with a particular focus on its potential use in seborrheic dermatitis and psoriasis. The aim of this paper is to provide a theoretical foundation and scientific support for the application of β‐glucan in the field of skin health, helping researchers refer to existing findings and explore areas that have not yet been fully investigated.

## 2 Characterization of β‐Glucan: The Impact of Molecular Structure, Physicochemical Properties, and Modifications on Its Function

### 2.1 Molecular Structure

**FIGURE 1:**
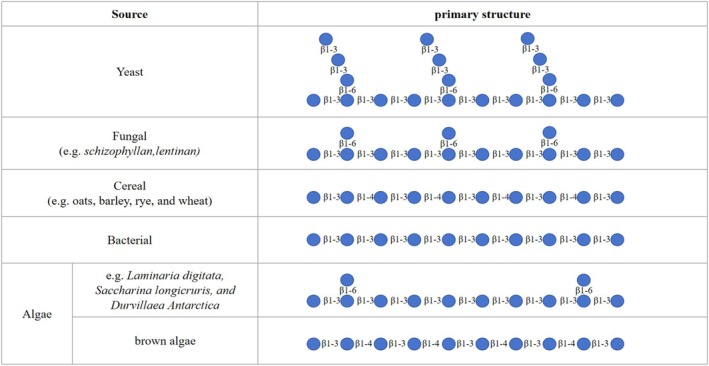
Structural features of β‐glucans. Adapted and modified from Caseiro et al. (2022), an open access article licensed under the Creative Commons Attribution (CC BY) license (https://creativecommons.org/licenses/by/4.0/).

The molecular structural characteristics of β‐glucan include molecular weight, glycosidic bond types, branching degree, and chain conformation (Kofuji et al. 2012). β‐glucans from different sources exhibit significant differences in these structural features, as shown in Figure 1.

β‐Glucans derived from fungi and yeast typically have a branched structure, consisting of a β‐(1 → 3)‐glucan backbone with side chains connected by β‐(1 → 6) glycosidic bonds (Samuelsen et al. 2011). Under the influence of hydrogen bonds, these backbones and side chains can form single or triple helix structures (Tiwari and Cummins 2009). Fungal β‐glucans exhibit β‐1,6 side chains of varying lengths, with those from mushrooms (e.g., *Lentinus edodes*) showing shorter β‐1,6 side chains (Han et al. 2020; Yuan et al. 2019).

Cereal β‐Glucans are unbranched linear polysaccharides, formed by β‐1,3 and β‐1,4 glycosidic bonds, and are referred to as “mixed‐linkage” β‐glucans (Wani et al. 2021). These are commonly found in oats, barley, rye, and wheat (Bohn and BeMiller 1995). Pure β‐(1,4)‐ and β‐(1,3)‐glucans are present in cellulose and gels, respectively, each with unique structural properties.

Bacterial β‐glucans, such as those derived from *Agribacterium biobaris*, typically have a linear β(1 → 3)‐D‐glucan structure (Zheng et al. 2017). In contrast, β‐glucans found in algae vary by species. For example, in brown algae, the cell wall contains insoluble (1 → 3) and (1 → 4)‐β‐D‐glucans (Lei et al. 2015). Meanwhile, the β‐glucans present in *Laminaria digitata* , *Saccharina longicruris*, and *Durvillaea antarctica* exhibit a β‐1,3 backbone with a few β‐1,6 glycosidic bonds and β‐1,6 glucose side‐chain branches (Sung et al. 2009).

#### 2.1.1 Conformation

The conformation of β‐glucan plays a crucial role in its biological function and is influenced by factors such as hydrogen bonding and molecular weight. β‐Glucan can adopt various structural forms, including single, double, or triple helices, random coils, aggregates, and rod‐like structures (Du et al. 2019). In fungal cell walls, high‐molecular‐weight β‐glucans predominantly exist in single or triple helix conformations, while low‐molecular‐weight β‐glucans tend to form random coils (Ohno et al. 1995). The triple helix conformation is particularly significant in immune signaling due to its enhanced ability to interact with cell receptors (Tejinder et al. 2000; Wu et al. 2021). Studies have demonstrated that (1 → 3)‐β‐D‐glucan in a triple helix conformation effectively inhibits S‐180 tumor growth, whereas the random coil form does not, and conversion of triple‐helix shiitake polysaccharides to a single‐chain structure leads to a marked reduction in anti‐tumor activity (Cheng et al. 2010; Yuan et al. 2019).

#### 2.1.2 Glycosidic Bond

The type of glycosidic bonds in β‐glucan is closely related to its immunological functions. Dectin‐1, a C‐type lectin‐like pattern recognition receptor, binds to glucans and induces innate immune responses against fungal pathogens (Meng et al. 2020). Studies show that Dectin‐1 has high specificity for β‐(1 → 3)‐D‐glucan, with binding affinity increasing as the polymer size and molecular weight (MW) rise. However, Dectin‐1 does not recognize glucans of cereals, as they contain mixed β‐(1 → 3) and β‐(1 → 4) linkages (Adams et al. 2008). Additionally, Dectin‐1 only interacts with β‐(1 → 3)‐D‐glucan oligosaccharides containing at least seven glucose units and a (1 → 6)‐β‐linked side chain at the non‐reducing end (Lowe et al. 2001).

Research also indicates that the immunological activity of cereal β‐glucans is partially dependent on the ratio of β‐(1,4)/β‐(1,3) linkages, and in some immune assays, this activity may activate the complement system through bypass pathways (Samuelsen et al. 2011).

#### 2.1.3 Branching Degree

The branching degree of β‐glucan also significantly impacts its biological activity (Tiwari and Cummins 2009). Studies indicate that β‐glucans with branching degrees between 0.04 and 0.75 exhibit bioactivity, with branching degrees between 0.2 and 0.33 often serving as effective immune modulators (Yuan et al. 2019). A study comparing the physiological activity of β‐glucans from three different mushrooms found that shiitake β‐glucan (branching degree 0.29) outperformed Chamsong‐I β‐glucan (branching degree 0.67) and cauliflower β‐glucan (branching degree 0.161). Activity further increased when the branching degree reached 32%, but decreased with higher branching degrees (Bae et al. 2013).

However, this trend is not absolute. For example, no significant difference in receptor binding affinity was observed between kelp polysaccharides (branching degree 0.1) and laminaria polysaccharides (branching degree 0.33), suggesting that the effect of branching degree on bioactivity may be modulated by other structural features (Han et al. 2020).

#### 2.1.4 MW

The MW of β‐glucan ranges from 10^2^ to 10^6^, with significant variation due to differences in source, extraction methods, and measurement techniques. Studies have shown that MW is closely related to β‐glucan's biological activity (Bohn and BeMiller 1995; Wani et al. 2021). In shiitake‐derived β‐glucan, low‐MW β‐glucans with a triple helix conformation exhibit significant anti‐tumor activity. This may be due to the shortened, rod‐like structure of the triple helix, which enhances rigidity and improves receptor binding, thereby boosting anti‐tumor effects (Zheng et al. 2017).

In contrast, low‐MW yeast β‐glucans are reported to have better antioxidant and immune activities (Ishimoto et al. 2018; Lei et al. 2015; Sung et al. 2009). However, for oat β‐glucan oligosaccharides, higher MW correlates with increased antioxidant activity, particularly in DPPH radical scavenging and lipid peroxidation inhibition, with similar trends observed in barley β‐glucan (Kofuji et al. 2012; Sun et al. 2020).

### 2.2 Physicochemical Properties

The molecular structural characteristics of β‐glucan directly determine its physicochemical properties, which in turn significantly influence its biological functions. Studies have shown that the functionality and activity of β‐glucan are closely related to its physicochemical properties, such as solubility, viscosity, and gelation characteristics (Du et al. 2019).

#### 2.2.1 Solubility

β‐glucan can be classified into water‐soluble and insoluble forms based on its solubility (Wu et al. 2021). The water solubility of β‐glucan primarily arises from its hydroxyl groups, which can form hydrogen bonds with water molecules, enhancing its hydrophilicity. Therefore, both water‐soluble and insoluble forms of β‐glucan effectively retain moisture (Tejinder et al. 2000). Studies show that the solubility of β‐glucan is closely linked to its molecular structure. For instance, branching structures or charged groups reduce intermolecular bonding, thereby increasing solubility, while linear chains, high MW, high polymerization, and regular arrangement generally decrease solubility (Cheng et al. 2010; Guo et al. 2017; Kim and White 2013; Yuan et al. 2019).

Specifically, β‐glucans with linear structures (e.g., some bacterial‐derived gel polysaccharides) are typically insoluble in water due to strong hydrogen bonds between molecules. In contrast, branched β‐glucans (e.g., shiitake polysaccharides) interact with hydroxyl groups in water, significantly enhancing solubility (Meng et al. 2020; Sousa et al. 2023). However, some high MW branched β‐glucans from yeast or fungi, despite having branched structures, remain insoluble due to fewer (1 → 6)‐β‐glycosidic linkages (Yuan et al. 2019).

Additionally, the solubility of β‐glucan is influenced by changes in its three‐dimensional structure. For example, when the molecular conformation shifts from an ordered triple helix to a loose triple helix or even a random coil, water solubility typically increases (Yan et al. 2020). Physical or chemical modifications can also effectively alter the solubility of β‐glucan, as described in Section 2.3.

The solubility of β‐glucan is a key factor affecting its biological activity. Research indicates that β‐glucans with different solubilities and structures exhibit significant variations in biological specificity and potency (Han et al. 2020). For instance, water‐soluble yeast β‐glucan enhances immune cell function by interacting with the complement system, while particulate β‐glucan activates dendritic cells (DCs) and macrophages through the Dectin‐1 receptor (Qi et al. 2011). Although Dectin‐1 can bind both soluble and particulate β‐glucans, signaling is only activated by particulate β‐glucans, making them ideal for inducing innate immune memory (Goodridge et al. 2011; Zhang et al. 2019).

In clinical applications, water‐soluble β‐glucan is widely used due to its ease of dissolution and administration, while particulate β‐glucans may be more effective in local immune modulation (Cleary et al. 1999). Compared to insoluble β‐glucan, water‐soluble β‐glucan has the advantage of reducing the risk of overactive immune responses and minimizing side effects. However, despite the strong immune‐stimulating effects of insoluble β‐glucan, its side effects and limitations in oral administration must be considered (Wu et al. 2021).

#### 2.2.2 Other Physicochemical Properties

In addition to solubility, other physicochemical properties of β‐glucan, such as viscosity and gelation characteristics, also play a crucial role in its functionality (Du et al. 2019; Lazaridou and Biliaderis 2007). Studies show that the viscosity of β‐glucan is closely related to its molecular weight. As the molecular weight increases, viscosity also increases. High molecular weight and viscosity β‐glucan can slow intestinal transit, reducing the absorption of glucose and sterols, thereby exerting cholesterol‐lowering and blood sugar‐lowering effects (Du et al. 2019; Sun et al. 2020; Wood 2007).

Furthermore, the gelation properties of β‐glucan enable it to serve as a carrier for bioactive compounds, effectively controlling their release (Lazaridou et al. 2015). This characteristic is essential for the development of pharmaceutical and nutritional formulations, as regulating the release rate can enhance the bioavailability and stability of active ingredients.

### 2.3 Modified β‐Glucan

The multiple hydroxyl groups in natural β‐glucan molecules form a tight triple helix structure, which limits its solubility and impedes its physiological functions in vivo. Modification of β‐glucan can significantly enhance its solubility and bioactivity, including antioxidant, anticancer, and immunomodulatory effects. Modification methods are categorized into physical, chemical, and biological modifications (Wang et al. 2017).

Physical modification involves techniques such as thermal degradation, irradiation, ultrasound treatment, and supercritical fluid technology, which break the macromolecular backbone without damaging the β‐glucan structure, thereby increasing solubility and functionality (Yuan et al. 2019). Chemical modification typically alters the structure of β‐glucan through processes like carboxymethylation, sulfation, phosphorylation, or acetylation (Edo et al. 2024). Biological modification mainly refers to enzyme‐catalyzed degradation of the polysaccharide. Compared to chemical modifications, biological modifications are more specific and have fewer side effects, but they tend to be more costly and less efficient (Zhang et al. 2022).

Studies show that various molecular modifications can enhance β‐glucan's antioxidant activity, with acetylation and sulfation being common methods, while phosphorylation is frequently used to improve anticancer activity (Li et al. 2016). Through modification, β‐glucan not only enhances its bioactivity but also expands its application potential in pharmaceuticals, nutrition, and skin health.

## 3 Biological Functions and Mechanisms of Action of β‐Glucans

### 3.1 Immunomodulatory Effects

**FIGURE 2:**
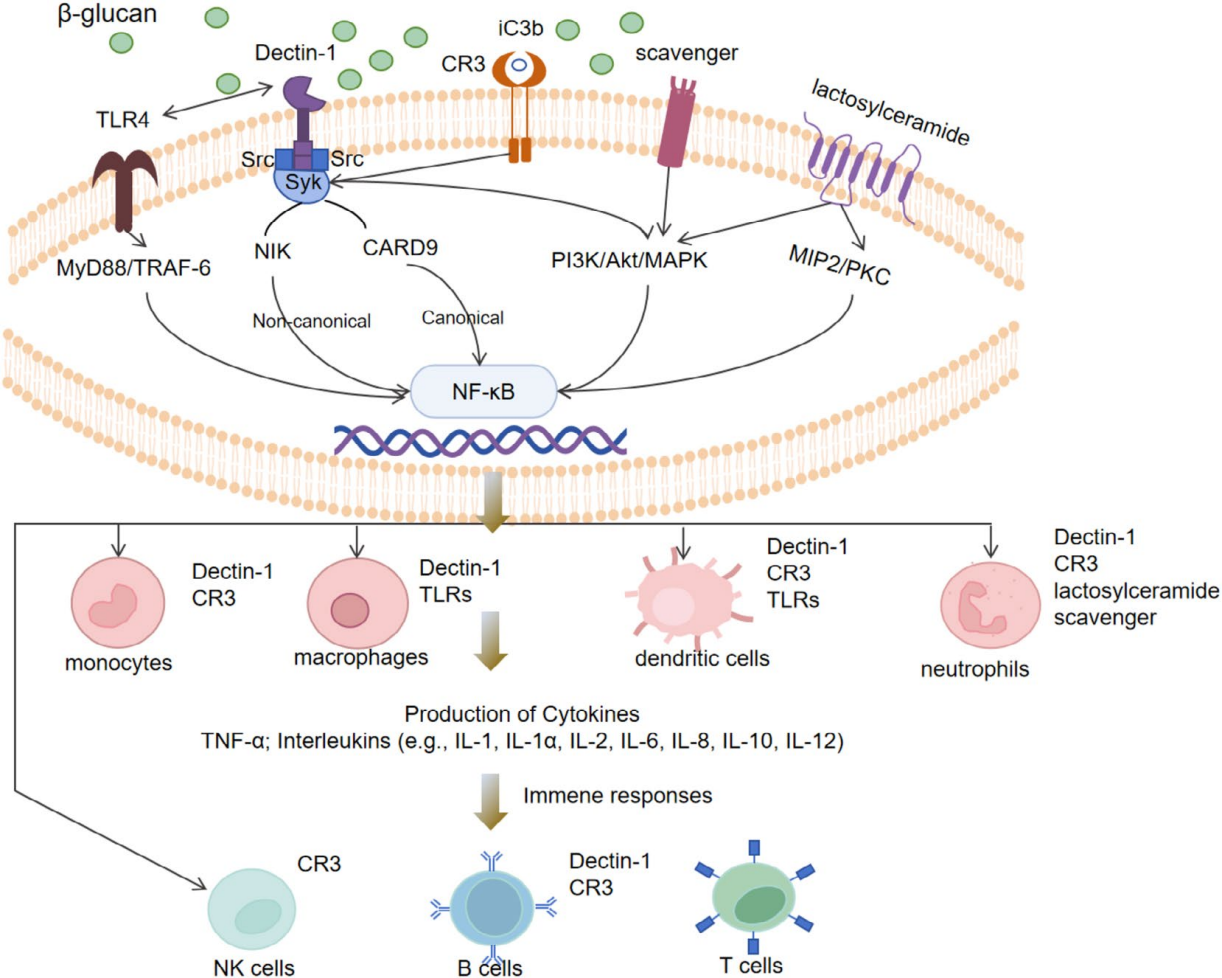
Mechanisms of the immunological effects of β‐Glucan. Adapted and modified from Dong et al. and Wu et al. (Dong et al. 2023; Wu et al. 2021). AKT, Protein Kinase B; CARD9, Caspase Recruitment Domain‐Containing Protein 9; CR3, complement receptor 3; iC3b, Inactive Complement Component 3b; IL, Interleukin; MAPK, Mitogen‐Activated Protein Kinase; MIP2, Macrophage Inflammatory Protein‐2; MYD88, Myeloid Differentiation Primary Response 88; NIK, NF‐κB‐Inducing Kinase; PI3K, Phosphoinositide 3‐Kinase; PKC, Protein Kinase C; Syk, Spleen Tyrosine Kinase; TLR4, Toll‐Like Receptor 4; TRAF6, TNF Receptor‐Associated Factor 6.

β‐glucan exerts significant immunomodulatory effects by binding to specific receptors on various immune cells, regulating both innate and adaptive immunity. These include effects on monocytes, macrophages, DCs, neutrophils, and natural killer (NK) cells (Chan et al. 2009). Key receptors for β‐glucan include Dectin‐1, complement receptor 3 (CR3), Toll‐like receptors (TLRs), scavenger receptors, and lactosylceramide (Wani et al. 2021). Binding to these receptors induces the production of cytokines and inflammatory mediators such as interleukins, TNF‐α, nitric oxide (NO), and hydrogen peroxide (H_2_O_2_) (Goodridge et al. 2009; Zhong et al. 2023), as shown in Figure 2.

Dectin‐1, a primary receptor for β‐glucan, is widely expressed on DCs, monocytes, macrophages, and neutrophils, playing a central role in β‐glucan recognition (Mata‐Martínez et al. 2022). Dectin‐1 signals through Src and Syk kinases, with Src phosphorylating ITAM‐like sequences to recruit Syk, which activates NF‐κB pathways via CARD9 and NIK, inducing pro‐inflammatory cytokine production and enhancing T cell responses (Goodridge et al. 2009; Peng et al. 2022). Dectin‐1 also cooperates with TLR4 to amplify NF‐κB activation, further boosting immune responses (Kanjan et al. 2017).

CR3 is an indirect receptor for β‐glucan, expressed on monocytes, neutrophils, NK cells, and lymphocytes (Chan et al. 2009). β‐glucan activates CR3 by binding to its lectin site, triggering the Syk‐PI3K signaling pathway to enhance neutrophil function (De Marco Castro et al. 2021; Wani et al. 2021). Additionally, iC3b, a ligand for CR3's I‐domain, further strengthens CR3‐mediated immune responses, including phagocytosis, degranulation, and antimicrobial activity when both β‐glucan and iC3b bind simultaneously (Bajic et al. 2013; Ross et al. 1999). Soluble β‐glucan modulates immunity via this pathway (Ross and Vĕtvicka 1993).

LacCer, a key neutral glycolipid found on neutrophil surfaces, activates Syk family kinases/PI3K signaling when bound to β‐glucan, further regulating immune responses (Cognigni et al. 2021). β‐glucan also interacts with scavenger receptors to induce MAPK activation and cytokine release (Vera et al. 2009).

### 3.2 Anti‐Inflammatory Effect

β‐glucan exerts anti‐inflammatory effects by modulating cytokines. Studies show that it regulates various inflammatory mediators, including NO, interleukins (ILs), TNF‐α, IFN‐γ, iNOS, and COX (Du et al. 2015). In various models, such as peritonitis, mouse THP‐1 cells, LPS‐induced macrophage models, and human skin cell models, β‐glucan demonstrates significant anti‐inflammatory activity (Ozanne et al. 2020; Queiroz et al. 2010; Wang et al. 2014; Xu et al. 2012).

Additionally, local application of β‐glucan also shows anti‐inflammatory effects. For example, oat β‐glucan combined with fermented probiotics reduces lymphocyte infiltration and dermal mast cell count in an Atopic dermatitis mouse model, suggesting potential for improving Atopic dermatitis (Kim et al. 2021). β‐glucans from other sources, such as *Agrocybe chaxingu*, can also inhibit LPS‐induced NO and COX‐2 expression, alleviating local inflammation (Lee et al. 2009).

Despite its diverse anti‐inflammatory actions, the exact mechanisms of β‐glucan remain unclear, and contradictions in existing studies complicate the understanding of its underlying mechanisms.

### 3.3 Antioxidant Effect

**FIGURE 3:**
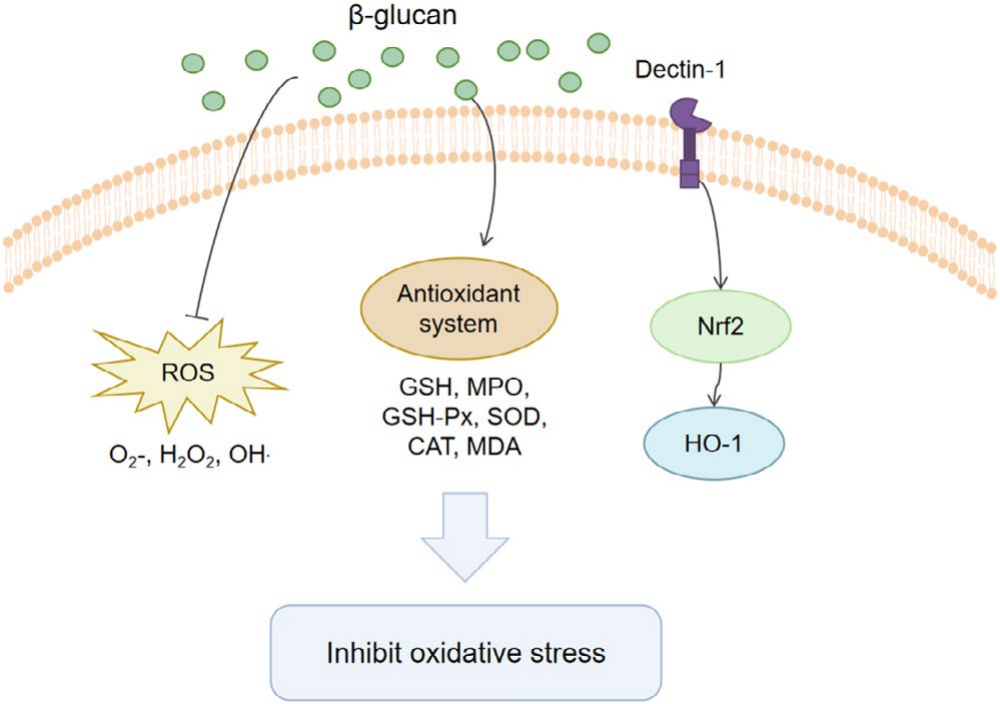
Mechanisms of the antioxidant effects of β‐Glucan. CAT, catalase; GSH, glutathione; GSH‐Px, glutathione peroxidase; H_2_O_2_, hydrogen peroxide; HO‐1, Heme Oxygenase‐1; MPO, myeloperoxidase; Nrf2, Nuclear Factor Erythroid 2‐Related Factor 2; O_2_^−^, superoxide; OH˙, hydroxyl radicals; ROS, reactive oxygen species; SOD, superoxide dismutase.

β‐glucan reduces oxidative damage, including lipid peroxidation, by scavenging reactive oxygen species (ROS), modulating the antioxidant system, and regulating oxidative stress‐mediated signaling pathways (Figure 3). ROS, including superoxide (O_2_^−^), hydrogen peroxide (H_2_O_2_), and hydroxyl radicals (OH˙), play crucial roles in cell signaling and immune defense. Excessive ROS production leads to oxidative stress and cellular damage, with OH˙ being the most active and potent oxidant among ROS (Zhang et al. 2016). Barley‐derived β‐glucan strongly inhibits HO (Kofuji et al. 2012). In vitro studies show that low molecular weight yeast β‐glucan not only scavenges HO·but also efficiently eliminates superoxide and DPPH (Lei et al. 2015). Other fungal‐derived β‐glucans also exhibit strong ROS‐scavenging abilities (Maity et al. 2015).

Numerous studies have demonstrated that β‐glucan reduces oxidative stress‐induced damage by modulating the body's antioxidant system. Both local and systemic applications of yeast β‐glucan significantly lower malondialdehyde (MDA, a final product of lipid peroxidation), maintain tissue glutathione (GSH) levels, and inhibit myeloperoxidase (MPO) activity in neutrophils, effectively protecting against oxidative damage. Notably, local application of β‐glucan offers significant protection in burn rat skin (Toklu et al. 2006). Additionally, β‐glucan from seaweed *Laminaria digitata* effectively inhibits ROS generation in human dermal fibroblasts and epidermal keratinocytes exposed to hydrogen peroxide and UVA radiation, showing strong antioxidant effects (Ozanne et al. 2020). Oral sulfated β‐Glucan from Saccharomyces cerevisiae significantly increases serum catalase (CAT) and glutathione peroxidase (GSH‐Px) activity in mice, while reducing MDA levels (Lei et al. 2015). Pleurotus ostreatus β‐glucan reduces conjugated dienes and glutathione levels in the colon and enhances superoxide dismutase (SOD), GSH‐Px, and glutathione reductase activity in the liver, significantly reducing precancerous lesions in the colon (Bobek and Galbavy 2001). High MW oat β‐glucan reduces lipid hydroperoxides (LOOH) and alleviates oxidative stress in the spleen in LPS‐induced colitis mice (Błaszczyk et al. 2015).

Furthermore, β‐glucan exerts its effects through oxidative stress‐related signaling pathways. A study has shown that β‐glucan from *Saccharomyces cerevisiae* mitigates LPS‐induced oxidative stress in RAW264.7 cells via the Dectin‐1/Nrf2/HO‐1 signaling pathway (Yu et al. 2021).

### 3.4 Barrier Repair

The skin barrier, primarily located in the stratum corneum, functions like a brick wall formed by keratinocytes and intercellular lipids, preventing water loss and external damage. Tight junctions and desmosomes further enhance barrier function, while fibroblasts in the dermis indirectly support it by synthesizing collagen (Hänel et al. 2013). Any abnormalities can lead to barrier dysfunction.

**FIGURE 4:**
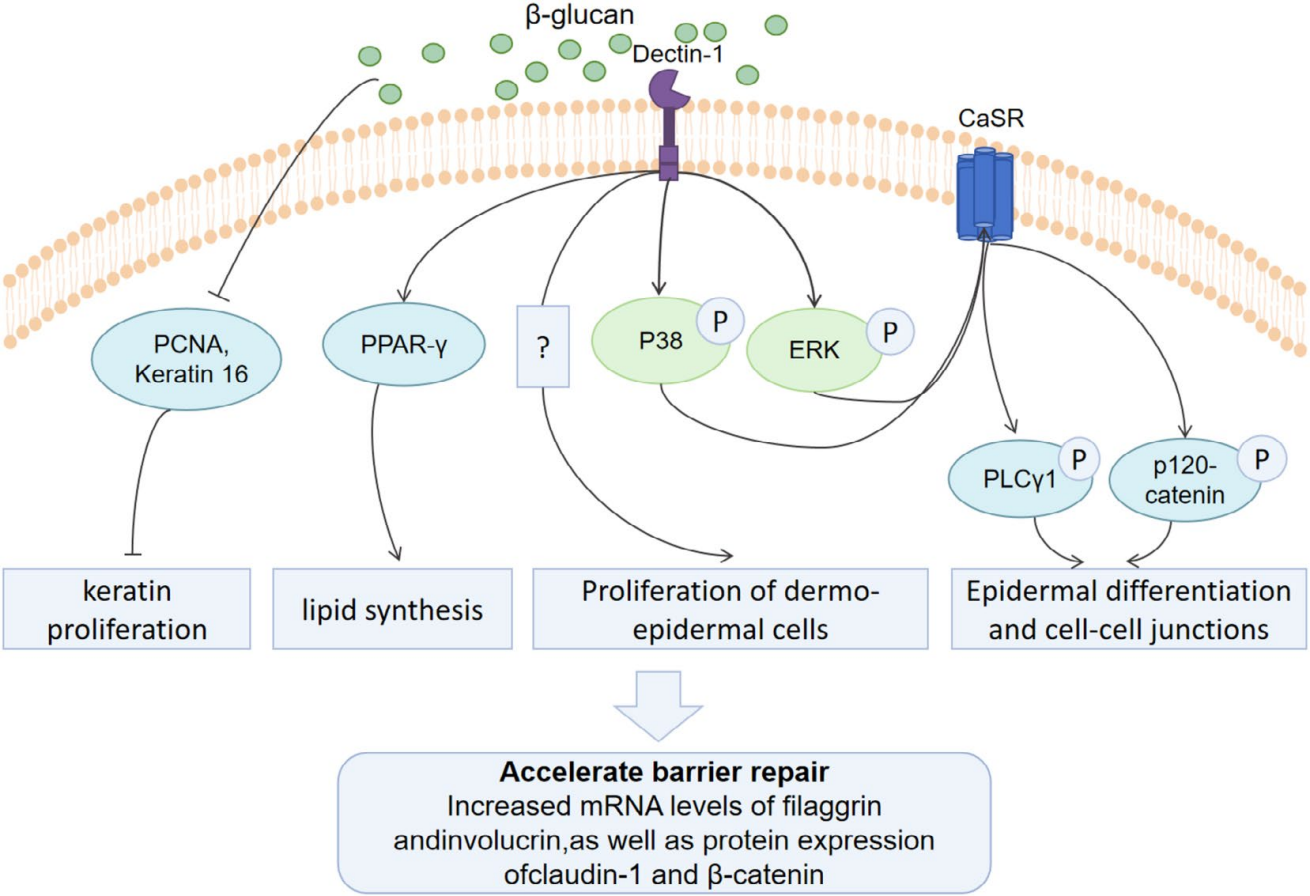
Mechanisms of the barrier repair of β‐Glucan. CaSR, calcium‐sensing receptor; ERK, Extracellular Signal‐Regulated Kinase; p38, p38 Mitogen‐Activated Protein Kinase; PCNA, proliferating cell nuclear antigen; PLCγ1, phospholipase Cγ1; PPAR‐γ, Peroxisome Proliferator‐Activated Receptor Gamma.

Studies indicate that β‐glucan plays a crucial role in barrier repair, as shown in Figure 4. Oat β‐glucan hydrogel, by activating the Dectin‐1 signaling pathway, effectively restores skin barrier function in mice, increasing mRNA levels of filaggrin and loricrin, as well as protein expression of claudin‐1 and β‐catenin. Mechanisms include: (1) Reduced expression of proliferating cell nuclear antigen (PCNA) and keratin 16 to decrease keratinocyte proliferation; (2) Phosphorylation of ERK and p38 MAPK to upregulate the calcium‐sensing receptor (CaSR) and phospholipase Cγ1 (PLCγ1), promoting epidermal differentiation and intercellular junctions; (3) Activation of PPAR‐γ to enhance lipid synthesis and accelerate barrier repair (Jing et al. 2024). Another study supports oat β‐glucan's role in barrier enhancement through the Dectin‐1‐ERK/p38‐CaSR pathway (Gao et al. 2021).

Additionally, gel polysaccharides promote keratinocyte proliferation and migration via Dectin‐1, improving wound healing (van den Berg et al. 2014). γ‐Propoxy‐sulfo‐lichenan (β‐1,3/1,4‐p‐d‐glucan) promotes the formation of terminal barriers in keratinocytes in a dose‐dependent manner (Esch et al. 2019).

β‐Glucan also enhances dermal fibroblast activity. Fungal β‐glucan increases L‐929 fibroblast proliferation and collagen synthesis In vitro, likely through macrophage‐released wound growth factors (Son et al. 2005). Barley β‐glucan accelerates human dermal fibroblast migration and enhances wound healing in mice (Fusté et al. 2019). Aureobasidium‐derived β‐glucan also stimulates fibroblast proliferation and migration, regulating TGF‐β1 for wound repair (Choi et al. 2016). β‐Glucan from *Schizophyllum commune* combined with polyvinyl alcohol (PVA) hydrogel promotes fibroblast migration and accelerates mouse wound healing, showing anti‐scar effects (Muthuramalingam et al. 2019).

### 3.5 Other Functions and Mechanisms

β‐Glucan exhibits significant moisturizing effects, primarily due to its multi‐helix molecular structure and hydrogen bonding between polar groups, forming a dense film that retains moisture and prevents water loss (Zhang et al. 2022a). Studies show that ultrasound‐degraded Cordyceps sinensis‐derived β‐glucan demonstrates superior moisture retention compared to its high‐molecular‐weight counterpart (Chen et al. 2014).

Additionally, β‐glucan's diverse bioactivities contribute to its UV‐protective and anti‐aging effects. Research indicates that yeast, fungal, bacterial, and modified β‐glucans exhibit in vitro and in vivo UV protection (Cheng et al. 2024; Li et al. 2023; Nanbu et al. 2011; Zulli et al. 1998). For example, carboxymethylated β‐glucan extracted from *Saccharomyces cerevisiae* effectively shields keratinocytes from UV‐A radiation (Zulli et al. 1998). *Agaricus blazei*‐derived β‐glucan significantly alleviates UVB‐induced skin damage in a HaCaT cell model (Cheng et al. 2024).

In recent years, β‐glucan has gained attention for its anti‐aging properties. β‐glucan from *Ganoderma lucidum* fruiting bodies shows whitening effects in vitro through anti‐tyrosinase and antioxidant activities, along with moderate inhibition of collagenase, elastase, and hyaluronidase, suggesting potential anti‐aging benefits (Vaithanomsat et al. 2022). In UV radiation‐induced cell models, *tremella* polysaccharide extract exhibited anti‐photoaging effects by enhancing collagen synthesis through the inhibition of matrix metalloproteinases (Choi and Kim 2021). Additionally, β‐glucan from *Agaricus blazei* demonstrated anti‐photoaging properties in UVA‐induced human fibroblast models by significantly increasing intracellular antioxidant enzyme levels and extracellular matrix proteins (such as COL‐I and ELN), while reducing the activity of metalloproteinases MMP‐1 and MMP‐9 (Di et al. 2024).

## 4 Clinical Applications of β‐Glucan in Skincare

β‐glucan, as a bioactive polysaccharide, has garnered significant attention in skincare due to its unique biological functions. It not only exhibits notable immunomodulatory effects but also plays a key role in anti‐inflammatory, antioxidant, barrier repair, moisturizing, and anti‐aging processes. These properties highlight the potential applications of β‐glucan in skincare.

### 4.1 Wound Healing

Delayed wound healing is one of the major therapeutic and economic issues in contemporary medicine (Schreml et al. 2010). Skin wound healing is a complex process, generally divided into three stages: inflammation, proliferation, and tissue remodeling (Witte and Barbul 1997). Multiple studies have shown that β‐glucan has potential in promoting wound healing.

Multiple studies have demonstrated the potential of β‐glucan in promoting wound healing. In a study tracking 12 patients with venous ulcers (13 ulcers in total, as one patient had two), local treatment with β‐glucan derived from baker's yeast for 30 days resulted in tissue biopsies showing epithelial proliferation and repair changes in 92.3% of the ulcers. An increase in fibroblasts and inflammatory cells was observed in all samples, accompanied by angiogenesis. After 30 days of treatment, the average ulcer area decreased by 11.3%, and by Day 90, the reduction averaged 55.23%. Notably, one patient's ulcer, which had not healed for 15 years, shrank by 67.8% after three months of treatment (Medeiros et al. 2012).

A randomized, double‐blind, double‐center, placebo‐controlled study evaluated the efficacy of soluble β‐1,3/1,6‐glucan (SBG) for local treatment of diabetic foot ulcers. Sixty patients with type 1 or type 2 diabetes received SBG or methylcellulose (as a control) three times a week in addition to standard care for up to 12 weeks. Fifty‐four patients completed the study, and the results showed that the SBG group tended to have a shorter median time to complete healing and a significant reduction in ulcer area during the first 6 weeks compared to the methylcellulose group. At Week 8, the healing rate in the SBG group was significantly higher (44% vs. 17%, *p* = 0.03), and the healing rate at Week 12 also favored SBG. SBG demonstrated good safety and tolerance (Zykova et al. 2014). Another study also confirmed the efficacy of β‐glucan in diabetic wound healing. Twenty‐two patients were administered 10 mg of β‐(1,3)‐glucan orally daily and applied wet β‐(1,3)‐glucan dressings topically. The results showed that most patients experienced rapid wound contraction and the formation of healthy granulation tissue. The average healing time was 10.8 weeks (ranging from 6 to 20 weeks), with no adverse events reported (Karaaslan et al. 2012).

Additionally, a study evaluated the impact of a β‐glucan‐containing skincare regimen on recovery after laser treatment. In 20 patients with facial acne scars undergoing CO_2_ fractional laser or 1565 nm non‐ablative laser treatment, the left side of the face was treated with the β‐glucan skincare regimen, while the right side served as the control. The results showed that the treatment group exhibited significantly improved hemoglobin indices on Day 7, as well as better skin hydration and reduced transepidermal water loss on both Day 7 and Day 14. Moreover, 63.2% of patients self‐reported better outcomes with the skincare regimen, with no significant side effects observed (Cao et al. 2021).

Other studies have also reported clinical effects of β‐glucan in promoting wound healing (Abedini et al. 2022; Thieme et al. 2016). These studies consistently demonstrate that β‐glucan exhibits significant potential in facilitating wound healing, providing a safe and effective treatment option.

### 4.2 Atopic Dermatitis

Atopic dermatitis is a common chronic inflammatory skin disease with a complex pathogenesis involving genetic factors, epidermal barrier defects, and immune response abnormalities (Sroka‐Tomaszewska and Trzeciak 2021). With the continuing rise in the prevalence of Atopic dermatitis, it has become a significant global health issue (Kellogg and Smogorzewski 2023).

A multicenter open‐label study evaluated the efficacy of β‐glucan cream in 105 Atopic dermatitis patients. The study required patients to apply a standard emollient systemically, while 0.25% β‐glucan cream was applied two to three times daily to the left side of the body. Results indicated that among the 80 patients who completed the study, itching and the severity of eczema significantly decreased, with significant reductions in the Visual Analog Scale (VAS) scores as well as the Eczema Area and Severity Index (EASI). (Jesenak et al. 2016).

Another study evaluated the effects of extracellular polysaccharides (EAP, containing 13% β‐1,3/1,6‐glucan) from Aureobasidium pullulans in patients with mild to moderate Atopic dermatitis. Sixty‐eight participants were randomly assigned to two groups: one group received 250 mg of EAP daily for 12 weeks, while the other received a placebo. The results showed that the EAP group had a significant reduction in Atopic dermatitis severity scores (SCORAD) compared to the placebo group, along with positive changes in serum interferon‐γ levels, skin hydration, and transepidermal water loss (Park et al. 2020).

Currently, clinical research on the application of β‐glucan in Atopic dermatitis remains limited. However, the existing findings suggest its potential in treating Atopic dermatitis, warranting further investigation to confirm its efficacy.

### 4.3 Anti‐Aging and Anti‐UV Damage

Clinical studies have shown that β‐glucan exhibits promising effects in anti‐aging and UV damage protection. For example, oat β‐glucan has been demonstrated to significantly reduce wrinkles. In a trial involving 27 participants, after 8 weeks of use, there were marked reductions in wrinkle height and depth, as well as skin roughness (Pillai et al. 2005). Additionally, daily use of β‐1,3/1,6‐glucan significantly improved skin elasticity (Calvani et al. 2020).

Two other randomized, double‐blind, placebo‐controlled clinical studies also confirmed the skin‐improving effects of β‐glucan and chitosan copolymer. In the first study, 13 participants with sensitive skin applied a formula containing 0.5%–2% β‐glucan twice daily for 6 weeks. No erythema was observed, and the water retention capacity of the stratum corneum and skin barrier function were enhanced. The second study involved 20 men showing signs of skin aging, who used a 1.5% β‐glucan formula twice daily for 16 weeks, resulting in significant improvements in skin firmness and hydration (Gautier et al. 2008).

Furthermore, a study on a β‐glucan cream evaluated its effects on skin discomfort caused by UVA/UVB exposure. The study included both short‐term and long‐term tests. The short‐term test showed that the cream significantly alleviated erythema within 24 h of UV exposure. The long‐term results indicated that after 30 days of use, skin hydration, brightness, elasticity, and antioxidant capacity were all improved (Schiano et al. 2021).

### 4.4 Other Skin Issues

β‐Glucan has clinical support for various skin conditions, including stretch marks, contact dermatitis, actinic keratosis, and foot xerosis. A study assessed the efficacy and tolerability of topical β‐glucan serum and lotion, 1565 nm non‐ablative fractional laser (NAFL), and their combination for treating stretch marks. Sixty‐four participants (128 unilateral abdomen sites) were randomly assigned to four groups: vehicle control, β‐glucan, NAFL + vehicle, and NAFL + β‐glucan, with a 12‐week follow‐up. NAFL was applied every 4 weeks, and the topical agents were used twice daily. Fifty‐six women (112 abdominal sites) completed the study. Results showed mild improvement in stretch marks with β‐glucan, more significant effects with NAFL, and potential enhancement when combined, with all treatments showing good tolerability (Cao et al. 2022).

In a double‐blind, placebo‐controlled study, 22 participants applied a formula containing 0.1% *Ginkgo biloba* extract and 0.5% carboxymethyl‐β‐1,3‐glucan twice daily on one forearm, with the other side receiving placebo, for 2 weeks. A contact allergen patch test was performed on Day 16. Results showed a significant reduction in skin reactions at the treatment site in 68.2% of participants (*p* = 0.037), suggesting the formula helped alleviate allergic contact dermatitis (Castelli et al. 1998).

A randomized, double‐blind, prospective pilot study investigated the effects and tolerability of yeast‐derived β‐glucan for treating actinic keratosis. Twenty participants applied either β‐glucan gel or placebo twice daily on both arms for 7 days, with evaluations at Weeks 1, 4, and 8. Both groups showed significant reductions in actinic keratosis lesions, though no significant differences between groups were observed, possibly due to the moisturizing effects of both treatments and the natural resolution of actinic keratosis, along with the short treatment duration (Tong and Barnetson 1996).

Additionally, the local application of chitosan‐glucan enhances stratum corneum hydration and improves foot xerosis in menopausal women with diabetes (Quatresooz et al. 2009). Other studies indicate that β‐glucan shows therapeutic potential for recurrent candidiasis, HPV‐related lesions, and epidermal repair processes (Pietrantoni et al. 2010).

## 5 Potential Applications of β‐Glucan in Seborrheic Dermatitis (SD) and Psoriasis (Ps)

SD and psoriasis Ps are common chronic inflammatory skin diseases, sharing similar clinical and pathological features, often presenting as red scaly plaques (Park et al. 2016). SD primarily affects areas rich in sebaceous glands (such as the scalp, face, and trunk), with a higher incidence during adolescence, peaking between the ages of 30 and 40 (Piquero‐Casals et al. 2019). Psoriasis is more commonly seen in high‐income regions and among the elderly (Parisi et al. 2020). Although their pathogenesis differs, both conditions share some common immunopathological features. Atopic dermatitis also exhibits similar immunopathological characteristics (Adalsteinsson et al. 2020). The occurrence of SD, Ps, and Atopic dermatitis is closely associated with *Malassezia* species, particularly in SD and Atopic dermatitis of the head and neck, where *Malassezia restricta* is the dominant species (Kim et al. 2016). Psoriatic skin is predominantly colonized by *Malassezia furfur* and *Malassezia restricta* (Liu et al. 2021). Studies have shown that these *Malassezia* species can stimulate the immune response in patients and exacerbate disease progression (Ashbee and Bond 2010). Abnormal host immune function may further promote *Malassezia* proliferation, thus worsening the disease (Adalsteinsson et al. 2020).

The treatment goals for these diseases generally include alleviating symptoms, controlling inflammation, repairing the skin barrier, preventing relapse, and improving quality of life. Treatment approaches often involve the use of immunosuppressants, such as topical corticosteroids and calcineurin inhibitors, primarily for anti‐inflammatory and immune‐modulating effects. At the same time, skincare products that promote barrier repair and topical moisturizers are also helpful in improving the condition (Clark et al. 2015; Ring et al. 2012; Torsekar and Gautam 2017).

β‐glucan, as a multi‐functional immune modulator, exhibits significant anti‐inflammatory and immunomodulatory properties. It can stimulate the production of various cytokines and exert effects through both immune and non‐immune mechanisms (Majtán and Jeseňák 2018). Moreover, β‐glucan can promote skin barrier repair and moisturization, and research has demonstrated its potential in alleviating Atopic dermatitis symptoms. Given the immunopathological similarities between SD, Ps, and Atopic dermatitis, as well as the role of β‐glucan in barrier repair and moisturization, it is hypothesized that β‐glucan may hold therapeutic potential for SD, Ps, and similar diseases. However, there are currently no specific studies on the use of β‐glucan for SD and Ps. Further research may open new directions for the treatment of these diseases.

## 6 Summary and Outlook

β‐glucan, as a natural polysaccharide, possesses significant biological activity and shows broad application potential in the field of skincare. Its main functions include immune modulation, antioxidant, anti‐inflammatory, skin barrier repair, and moisturizing, all of which have been validated through numerous clinical and basic research studies. Notably, beta‐glucan has demonstrated good efficacy in the treatment of skin issues such as wound healing, protection against ultraviolet damage, photoaging, and Atopic dermatitis. This paper also analyzes the therapeutic potential of beta‐glucan in skin disorders like SD and Ps, which, in terms of immunopathology, share similarities with Atopic dermatitis.

Despite the progress made in the application of β‐glucan in skincare, several key challenges remain in fully unlocking its potential. First, the mechanism of action of β‐glucan still requires further exploration. A deeper understanding of its biological effects on the skin will provide theoretical support for its scientific application and maximize its potential benefits. Second, the sources of β‐glucan are diverse, and its structural variations lead to different biological activities. Future research should focus on investigating the specific biological functions of β‐glucans from different sources and molecular structures, and determining the optimal application methods in skincare. Additionally, the formulation of β‐glucan needs further optimization, particularly in enhancing its solubility and bioavailability, to improve its efficacy in topical skin applications. On the other hand, modification techniques, such as physical, chemical, or biological modifications, offer effective ways to enhance the performance of β‐glucan in skincare.

In conclusion, the application potential of β‐glucan in skincare is vast. As research continues to advance, its application prospects in skin health will become even broader, providing more scientific and effective treatment solutions for various skin problems.

## Author Contributions

**Xiaoyue Feng:** conceptualization (equal), investigation (equal), methodology (equal), validation (equal), writing – original draft (lead), writing – review and editing (lead). **Jianli Shang:** conceptualization (equal), investigation (equal), methodology (equal). **Yuhui Wang:** conceptualization (equal), investigation (equal), methodology (equal). **Yong Chen:** supervision (equal), validation (equal). **Youting Liu:** conceptualization (equal), methodology (equal), supervision (equal).

## Conflicts of Interest

The authors declare no conflicts of interest.

## Data Availability

The authors have nothing to report.

## References

[fsn370212-bib-0001] Abedini, F. , S. R. Mohammadi , M. Dahmardehei , et al. 2022. “Enhancing of Wound Healing in Burn Patients Through *Candida Albicans* β‐Glucan.” Journal of Fungi 8, no. 3: 263. 10.3390/jof8030263. 35330265 PMC8949177

[fsn370212-bib-0002] Adalsteinsson, J. A. , S. B. Kaushik , S. Muzumdar , E. Guttman‐Yassky , and J. P. Ungar . 2020. “An Update on the Microbiology, Immunology and Genetics of Seborrheic Dermatitis.” Experimental Dermatology 29, no. 5: 481–489. 10.1111/exd.14091. 32125725

[fsn370212-bib-0003] Adams, E. L. , P. J. Rice , B. Graves , et al. 2008. “Differential High‐Affinity Interaction of Dectin‐1 With Natural or Synthetic Glucans Is Dependent Upon Primary Structure and Is Influenced by Polymer Chain Length and Side‐Chain Branching.” Journal of Pharmacology and Experimental Therapeutics 325, no. 1: 115–123. 10.1124/jpet.107.133124. 18171906

[fsn370212-bib-0004] Ashbee, H. R. , and R. Bond . 2010. “Malassezia Species and Immunity: Host–Pathogen Interactions.” In Malassezia and the Skin, edited by T. Boekhout , P. Mayser , E. Guého‐Kellermann , and A. Velegraki . Springer. 10.1007/978-3-642-03616-3_5.

[fsn370212-bib-0005] Bae, I. Y. , H. Kim , H. J. Yoo , et al. 2013. “Correlation of Branching Structure of Mushroom β‐Glucan With Its Physiological Activities.” Food Research International 51, no. 1: 195–200. 10.1016/j.foodres.2012.12.008.

[fsn370212-bib-0006] Bajic, G. , L. Yatime , R. B. Sim , T. Vorup‐Jensen , and G. R. Andersen . 2013. “Structural Insight on the Recognition of Surface‐Bound Opsonins by the Integrin I Domain of Complement Receptor 3.” Proceedings of the National Academy of Sciences of the United States of America 110, no. 41: 16426–16431. 10.1073/pnas.1311261110. 24065820 PMC3799375

[fsn370212-bib-0007] Błaszczyk, K. , J. Wilczak , J. Harasym , et al. 2015. “Impact of Low and High Molecular Weight Oat Beta‐Glucan on Oxidative Stress and Antioxidant Defense in Spleen of Rats With LPS Induced Enteritis.” Food Hydrocolloids 51: 272–280. 10.1016/j.foodhyd.2015.05.025. 25520199

[fsn370212-bib-0008] Bobek, P. , and S. Galbavy . 2001. “Effect of Pleuran (Beta‐Glucan From Pleurotus Ostreatus) on the Antioxidant Status of the Organism and on Dimethylhydrazine‐Induced Precancerous Lesions in Rat Colon.” British Journal of Biomedical Science 58, no. 3: 164–168. 11575739

[fsn370212-bib-0009] Bohn, J. A. , and J. N. BeMiller . 1995. “(1→3)‐β‐d‐Glucans as Biological Response Modifiers: A Review of Structure‐Functional Activity Relationships.” Carbohydrate Polymers 28: 3–14.

[fsn370212-bib-0010] Calvani, F. , E. Bartoletti , G. Folchitto , S. Santini , M. Fontevecchia , and A. Alhadeff . 2020. “Innovation in Skin Regeneration: Novel Approaches With β‐1,3/1,6‐Glucan‐Based Treatments.” Recent Progress in Materials 2, no. 2: 1–7. 10.21926/rpm.2002011.

[fsn370212-bib-0011] Cao, Y. , P. Wang , X. Liu , and G. Zhang . 2022. “Improving the Outcome of Treating Striae Gravidarum by Combined Therapies Using Topical β‐Glucan and 1565‐Nm Non‐Ablative Fractional Laser: A Prospective Randomized Vehicle‐Controlled Parallel Group Study.” Journal of Dermatology 49, no. 9: 829–836. 10.1111/1346-8138.16417. 35570397

[fsn370212-bib-0012] Cao, Y. , P. Wang , G. Zhang , C. Hu , H. Zhang , and X. Wang . 2021. “Administration of Skin Care Regimens Containing β‐Glucan for Skin Recovery After Fractional Laser Therapy: A Split‐Face, Double‐Blinded, Vehicle‐Controlled Study.” Journal of Cosmetic Dermatology 20, no. 6: 1756–1762. 10.1111/jocd.13798. 33128496

[fsn370212-bib-0013] Caseiro, C. , J. N. R. Dias , C. M. G. de Andra Fontes , and P. Bule . 2022. “From Cancer Therapy to Winemaking: The Molecular Structure and Applications of β‐Glucans and β‐1, 3‐Glucanases.” Ijms 23, no. 6: 3156. 10.3390/ijms23063156. 35328577 PMC8949617

[fsn370212-bib-0014] Castelli, D. , L. Colin , E. Camel , and G. Ries . 1998. “Pretreatment of Skin With a *Ginkgo biloba* Extract/Sodium Carboxymethyl‐Beta‐1,3‐Glucan Formulation Appears to Inhibit the Elicitation of Allergic Contact Dermatitis in Man.” Contact Dermatitis 38, no. 3: 123–126. 10.1111/j.1600-0536.1998.tb05676.x. 9536401

[fsn370212-bib-0015] Chan, G. C.‐F. , W. K. Chan , and D. M.‐y. Sze . 2009. “The Effects of β‐Glucan on Human Immune and Cancer Cells.” Journal of Hematology & Oncology 2, no. 1: 25. 10.1186/1756-8722-2-25. 19515245 PMC2704234

[fsn370212-bib-0016] Chen, X. , K. C. Siu , Y. C. Cheung , and J. Y. Wu . 2014. “Structure and Properties of a (1→3)‐β‐D‐Glucan From Ultrasound‐Degraded Exopolysaccharides of a Medicinal Fungus.” Carbohydrate Polymers 106: 270–275. 10.1016/j.carbpol.2014.02.040. 24721078

[fsn370212-bib-0017] Cheng, W. , F. Di , L. Li , and C. Pu . 2024. “Anti‐Photodamage Effect of Agaricus Blazei Murill Polysaccharide on UVB‐Damaged HaCaT Cells.” International Journal of Molecular Sciences 25, no. 9: 4676. 10.3390/ijms25094676. 38731895 PMC11083510

[fsn370212-bib-0018] Cheng, W.‐L. , J. Chen , D. Liu , X. Ye , and F. Ke . 2010. “Impact of Ultrasonic Treatment on Properties of Starch Film‐Forming Dispersion and the Resulting Films.” Carbohydrate Polymers 81, no. 3: 707–711. 10.1016/j.carbpol.2010.03.043.

[fsn370212-bib-0019] Choi, J.‐H. , and B.‐G. Kim . 2021. “Improvement of Skin Photoaging by Polysaccharide Extract Derived From Tremella Fuciformis (White Jelly Mushroom).” Natural Product Sciences 27, no. 4: 300–306. 10.20307/nps.2021.27.4.300.

[fsn370212-bib-0020] Choi, J. S. , J. W. Kim , G.‐w. Jung , et al. 2016. “Effect of a β‐Glucan From Aureobasidium on TGF‐β1‐Modulated In Vitro Dermal Wound Repair.” Toxicology and Environmental Health Sciences 8, no. 1: 12–18. 10.1007/s13530-016-0257-1.

[fsn370212-bib-0021] Clark, G. , S. M. Pope , and K. A. Jaboori . 2015. “Diagnosis and Treatment of Seborrheic Dermatitis.” American Family Physician 91, no. 3: 185–190. 25822272

[fsn370212-bib-0022] Cleary, J. A. , G. E. Kelly , and A. J. Husband . 1999. “The Effect of Molecular Weight and Beta‐1,6‐Linkages on Priming of Macrophage Function in Mice by (1,3)‐Beta‐D‐Glucan.” Immunology and Cell Biology 77, no. 5: 395–403. 10.1046/j.1440-1711.1999.00848.x. 10540205

[fsn370212-bib-0023] Cognigni, V. , N. Ranallo , F. Tronconi , F. Morgese , and R. Berardi . 2021. “Potential Benefit of β‐Glucans as Adjuvant Therapy in Immuno‐Oncology: A Review.” Exploration of Targeted Anti‐Tumor Therapy 2, no. 2: 122–138. 10.37349/etat.2021.00036. 36046144 PMC9400766

[fsn370212-bib-0024] De Marco Castro, E. , P. C. Calder , and H. M. Roche . 2021. “β‐1,3/1,6‐Glucans and Immunity: State of the Art and Future Directions.” Molecular Nutrition & Food Research 65, no. 1: e1901071. 10.1002/mnfr.201901071. 32223047 PMC7816268

[fsn370212-bib-0025] Di, F. , W. Cheng , L. Li , et al. 2024. “Identifying a Role of Polysaccharides From Agaricus Blazei Murill in Combating Skin Photoaging: The Effect of Antioxidants on Fibroblast Behavior.” Fermentation 10, no. 6: 292. 10.3390/fermentation10060292.

[fsn370212-bib-0026] Di Luzio, N. R. , and S. J. Riggi . 1970. “The Effects of Laminarin, Sulfated Glucan and Oligosaccharides of Glucan on Reticuloendothelial Activity.” Journal of the Reticuloendothelial Society 8, no. 5: 465–473. 5471976

[fsn370212-bib-0027] Dong, Q.‐Q. , Q. Wu , Y. Lu , et al. 2023. “Exploring β‐Glucan as a Micro‐Nano System for Oral Delivery Targeted the Colon.” International Journal of Biological Macromolecules 253, no. Pt 6: 127360. 10.1016/j.ijbiomac.2023.127360. 37827417

[fsn370212-bib-0028] Du, B. , C.‐y. Lin , Z.‐x. Bian , and B. Xu . 2015. “An Insight Into Anti‐Inflammatory Effects of Fungal Beta‐Glucans.” Trends in Food Science & Technology 41, no. 1: 49–59. 10.1016/j.tifs.2014.09.002.

[fsn370212-bib-0029] Du, B. , M. Meenu , H. Liu , and B. Xu . 2019. “A Concise Review on the Molecular Structure and Function Relationship of β‐Glucan.” International Journal of Molecular Sciences 20, no. 16: 4032. 10.3390/ijms20164032. 31426608 PMC6720260

[fsn370212-bib-0030] Edo, G. I. , W. Ndudi , R. S. Makia , et al. 2024. “Beta‐Glucan: An Overview in Biological Activities, Derivatives, Properties, Modifications and Current Advancements in Food, Health and Industrial Applications.” Process Biochemistry 147: 347–370. 10.1016/j.procbio.2024.09.011.

[fsn370212-bib-0031] Esch, S. , M. Gottesmann , and A. Hensel . 2019. “γ‐Propoxy‐Sulfo‐Lichenan Induces in Vitro Cell Differentiation of Human Keratinocytes.” Molecules 24, no. 3: 574. 10.3390/molecules24030574. 30764551 PMC6384931

[fsn370212-bib-0032] Fusté, N. P. , M. Guasch , P. V. Guillen , et al. 2019. “Barley β‐Glucan Accelerates Wound Healing by Favoring Migration Versus Proliferation of Human Dermal Fibroblasts.” Carbohydrate Polymers 210: 389–398. 10.1016/j.carbpol.2019.01.090. 30732776

[fsn370212-bib-0033] Gao, S. , Y. Chen , J. Zhao , et al. 2021. “Oat β‐Glucan Ameliorates Epidermal Barrier Disruption by Upregulating the Expression of CaSR Through Dectin‐1‐Mediated ERK and p38 Signaling Pathways.” International Journal of Biological Macromolecules 185: 876–889. 10.1016/j.ijbiomac.2021.07.002. 34237364

[fsn370212-bib-0034] Gautier, S. , E. Xhauflaire‐Uhoda , P. Gonry , and G. E. Piérard . 2008. “Chitin‐Glucan, a Natural Cell Scaffold for Skin Moisturization and Rejuvenation.” International Journal of Cosmetic Science 30, no. 6: 459–469. 10.1111/j.1468-2494.2008.00470.x. 19099547

[fsn370212-bib-0035] Goodridge, H. S. , C. N. Reyes , C. A. Becker , et al. 2011. “Activation of the Innate Immune Receptor Dectin‐1 Upon Formation of a Phagocytic Synapse.” Nature 472, no. 7344: 471–475. 10.1038/nature10071. 21525931 PMC3084546

[fsn370212-bib-0036] Goodridge, H. S. , A. J. Wolf , and D. M. Underhill . 2009. “β‐Glucan Recognition by the Innate Immune System.” Immunological Reviews 230: 38–50. 19594628 10.1111/j.1600-065X.2009.00793.x PMC6618291

[fsn370212-bib-0037] Guo, M. Q. , X. Hu , C. Wang , and L.‐z. Ai . 2017. Polysaccharides: Structure and Solubility. Intechopen.

[fsn370212-bib-0038] Han, B. , K. Baruah , E. Cox , D. Vanrompay , and P. Bossier . 2020. “Structure‐Functional Activity Relationship of β‐Glucans From the Perspective of Immunomodulation: A Mini‐Review.” Frontiers in Immunology 11: 658. 10.3389/fimmu.2020.00658. 32391005 PMC7188827

[fsn370212-bib-0039] Hänel, K. H. , C. Cornelissen , B. Lüscher , and J. M. Baron . 2013. “Cytokines and the Skin Barrier.” International Journal of Molecular Sciences 14, no. 4: 6720–6745. 10.3390/ijms14046720. 23531535 PMC3645662

[fsn370212-bib-0040] Ishimoto, Y. , K. I. Ishibashi , D. Yamanaka , et al. 2018. “Production of Low‐Molecular Weight Soluble Yeast β‐Glucan by an Acid Degradation Method.” International Journal of Biological Macromolecules 107, no. Pt B: 2269–2278. 10.1016/j.ijbiomac.2017.10.094. 29051097

[fsn370212-bib-0041] Jesenak, M. , S. Urbancek , J. Majtan , P. Banovcin , and J. Hercogova . 2016. “β‐Glucan‐Based Cream (Containing Pleuran Isolated From Pleurotus Ostreatus) in Supportive Treatment of Mild‐To‐Moderate Atopic Dermatitis.” Journal of Dermatological Treatment 27, no. 4: 351–354. 10.3109/09546634.2015.1117565. 26654776

[fsn370212-bib-0042] Jing, R. , M. Fu , Y. Huang , K. Zhang , J. Ye , and F. Gong . 2024. “Oat β‐Glucan Repairs the Epidermal Barrier by Upregulating the Levels of Epidermal Differentiation, Cell‐Cell Junctions and Lipids via Dectin‐1.” British Journal of Pharmacology 181, no. 11: 1596–1613. 10.1111/bph.16306. 38124222

[fsn370212-bib-0043] Kanjan, P. , N. M. Sahasrabudhe , B. J. d. Haan , and P. D. Vos . 2017. “Immune Effects of β‐Glucan Are Determined by Combined Effects on Dectin‐1, TLR2, 4 and 5.” Journal of Functional Foods 37: 433–440. 10.1016/j.jff.2017.07.061.

[fsn370212-bib-0044] Karaaslan, O. , Y. Kankaya , N. Sungur , et al. 2012. “Case Series of Topical and Orally Administered β‐Glucan for the Treatment of Diabetic Wounds: Clinical Study.” Journal of Cutaneous Medicine and Surgery 16, no. 3: 180–186. 10.1177/120347541201600308. 22713441

[fsn370212-bib-0045] Kellogg, C. , and J. M. Smogorzewski . 2023. “Update on Atopic Dermatitis.” Advances in Pediatrics 70, no. 1: 157–170. 10.1016/j.yapd.2023.03.006. 37422293

[fsn370212-bib-0046] Kim, H. J. , and P. J. White . 2013. “Impact of the Molecular Weight, Viscosity, and Solubility of β‐Glucan on In Vitro Oat Starch Digestibility.” Journal of Agricultural and Food Chemistry 61, no. 13: 3270–3277. 10.1021/jf305348j. 23469761

[fsn370212-bib-0047] Kim, S.‐Y. , S. H. Kim , S. N. Kim , et al. 2016. “Isolation and Identification of Malassezia Species From Chinese and Korean Patients With Seborrheic Dermatitis and In Vitro Studies on Their Bioactivity on Sebaceous Lipids and IL‐8 Production.” Mycoses 59, no. 5: 274–280. 10.1111/myc.12456. 26786542

[fsn370212-bib-0048] Kim, Y. H. , M. S. Kang , T. H. Kim , et al. 2021. “Anti‐Inflammatory and Immune Modulatory Effects of Synbio‐Glucan in an Atopic Dermatitis Mouse Model.” Nutrients 13, no. 4: 1090. 10.3390/nu13041090. 33810608 PMC8067118

[fsn370212-bib-0049] Kofuji, K. , A. Aoki , K. Tsubaki , M. Konishi , T. Isobe , and Y. Murata . 2012. “Antioxidant Activity of β‐Glucan.” ISRN Pharmaceutics: 125864. 22500243 10.5402/2012/125864 PMC3302110

[fsn370212-bib-0050] Lazaridou, A. , and C. G. Biliaderis . 2007. “Molecular Aspects of Cereal β‐Glucan Functionality: Physical Properties, Technological Applications and Physiological Effects.” Journal of Cereal Science 46: 101–118.

[fsn370212-bib-0051] Lazaridou, A. , K. Kritikopoulou , and C. G. Biliaderis . 2015. “Barley β‐Glucan Cryogels as Encapsulation Carriers of Proteins: Impact of Molecular Size on Thermo‐Mechanical and Release Properties.” Bioactive Carbohydrates and Dietary Fibre 6: 99–108.

[fsn370212-bib-0052] Lee, B. R. , S. Y. Kim , D. W. Kim , et al. 2009. “Agrocybe Chaxingu Polysaccharide Prevent Inflammation Through the Inhibition of COX‐2 and NO Production.” BMB Reports 42, no. 12: 794–799. 10.5483/bmbrep.2009.42.12.794. 20044950

[fsn370212-bib-0053] Lei, N. , M. Wang , L. Zhang , et al. 2015. “Effects of Low Molecular Weight Yeast β‐Glucan on Antioxidant and Immunological Activities in Mice.” International Journal of Molecular Sciences 16, no. 9: 21575–21590. 10.3390/ijms160921575. 26370978 PMC4613268

[fsn370212-bib-0054] Li, S. , Q. Xiong , X. Lai , et al. 2016. “Molecular Modification of Polysaccharides and Resulting Bioactivities.” Comprehensive Reviews in Food Science and Food Safety 15, no. 2: 237–250. 10.1111/1541-4337.12161. 33371599

[fsn370212-bib-0055] Li, Z. , Y.‐l. Huang , J. Zhang , D. Mi , and W.‐W. Zhou . 2023. “Ultrasound Stimulated Production of Exopolysaccharide With Anti‐UV Radiation Activity by Increasing Cell Permeability of *Paenibacillus polymyxa* .” Process Biochemistry 126: 252–259. 10.1016/j.procbio.2023.01.010.

[fsn370212-bib-0056] Liu, X. , Q. Cai , H. Y. Yang , Z.‐q. Gao , and L. Yang . 2021. “Distribution of Malassezia Species on the Skin of Patients With Psoriasis.” Journal de Mycologie Médicale 31, no. 2: 101111. 10.1016/j.mycmed.2021.101111. 33454614

[fsn370212-bib-0057] Lowe, E. , P. Rice , T. Ha , et al. 2001. “A (1–>3)‐Beta‐D‐Linked Heptasaccharide Is the Unit Ligand for Glucan Pattern Recognition Receptors on Human Monocytes.” Microbes and Infection 3, no. 10: 789–797. 10.1016/s1286-4579(01)01436-8. 11580973

[fsn370212-bib-0058] Maity, P. , I. K. Sen , P. K. Maji , et al. 2015. “Structural, Immunological, and Antioxidant Studies of β‐Glucan From Edible Mushroom Entoloma Lividoalbum.” Carbohydrate Polymers 123: 350–358. 10.1016/j.carbpol.2015.01.051. 25843868

[fsn370212-bib-0059] Majtán, J. , and M. Jeseňák . 2018. “β‐Glucans: Multi‐Functional Modulator of Wound Healing.” Molecules: A Journal of Synthetic Chemistry and Natural Product Chemistry 23: 806. 29614757 10.3390/molecules23040806 PMC6017669

[fsn370212-bib-0060] Mata‐Martínez, P. , M. Bergón‐Gutiérrez , and C. Del Fresno . 2022. “Dectin‐1 Signaling Update: New Perspectives for Trained Immunity.” Frontiers in Immunology 13: 812148. 10.3389/fimmu.2022.812148. 35237264 PMC8882614

[fsn370212-bib-0061] Medeiros, S. D. V. , S. L. Cordeiro , J. E. C. Cavalcanti , et al. 2012. “Effects of Purified *Saccharomyces Cerevisiae* (1→3)‐β‐Glucan on Venous Ulcer Healing.” International Journal of Molecular Sciences 13, no. 7: 8142–8158. 10.3390/ijms13078142. 22942695 PMC3430226

[fsn370212-bib-0062] Meng, Y. , F. Lyu , and X. Xu . 2020. “Recent Advances in Chain Conformation and Bioactivities of Triple‐Helix Polysaccharides.” Biomacromolecules 21, no. 5: 1653–1677. 10.1021/acs.biomac.9b01644. 31986015

[fsn370212-bib-0063] Muthuramalingam, K. , S. I. Choi , C. Hyun , Y. M. Kim , and M. Cho . 2019. “β‐Glucan‐Based Wet Dressing for Cutaneous Wound Healing.” Advances in Wound Care 8, no. 4: 125–135. 10.1089/wound.2018.0843. 31737411 PMC6855293

[fsn370212-bib-0064] Nanbu, T. , T. Matsuta , H. Sakagami , J. Shimada , J. Maki , and T. Makino . 2011. “Anti‐UV Activity of Lentinus Edodes Mycelia Extract (LEM).” In Vivo 25, no. 5: 733–740. 21753126

[fsn370212-bib-0065] Ohno, N. , N. N. Miura , N. Chiba , Y. Adachi , and T. Yadomae . 1995. “Comparison of the Immunopharmacological Activities of Triple and Single‐Helical Schizophyllan in Mice.” Biological & Pharmaceutical Bulletin 18, no. 9: 1242–1247. 8845814 10.1248/bpb.18.1242

[fsn370212-bib-0066] Ozanne, H. , H. Toumi , B. Roubinet , et al. 2020. “Laminarin Effects, a β‐(1,3)‐Glucan, on Skin Cell Inflammation and Oxidation.” Cosmetics 7: 66.

[fsn370212-bib-0067] Parisi, R. , I. Y. K. Iskandar , E. Kontopantelis , M. Augustin , C. E. M. Griffiths , and D. M. Ashcroft . 2020. “National, Regional, and Worldwide Epidemiology of Psoriasis: Systematic Analysis and Modelling Study.” BMJ 369: m1590. 10.1136/bmj.m1590. 32467098 PMC7254147

[fsn370212-bib-0068] Park, H.‐S. , M. Kang , Y.‐M. Kim , et al. 2020. “A Clinical Study on the Efficacy and Safety of the Exopolymers From Aureobasidium Pullulans (EAP) in Subjects With Mild‐To‐Moderate Atopic Dermatitis.” Toxicology and Environmental Health Sciences 12, no. 1: 31–43. 10.1007/s13530-020-00040-y.

[fsn370212-bib-0069] Park, J.‐H. , Y. J. Park , S. K. Kim , et al. 2016. “Histopathological Differential Diagnosis of Psoriasis and Seborrheic Dermatitis of the Scalp.” Annals of Dermatology 28, no. 4: 427–432. 10.5021/ad.2016.28.4.427. 27489423 PMC4969470

[fsn370212-bib-0070] Peng, Y. , Y. Chen , J. Ma , et al. 2022. “Role and Mechanism of the Dectin‐1‐Mediated Syk/NF‐κB Signaling Pathway in Talaromyces Marneffei Infection.” Experimental and Therapeutic Medicine 23, no. 1: 84. 10.3892/etm.2021.11007. 34938366 PMC8688926

[fsn370212-bib-0071] Pietrantoni, E. , F. Signore , G. Berardi , F. Donadio , and C. Donadio . 2010. “Role of Beta‐Glucan in the Treatment of Recurrent Candidiasis and HPV‐Correlated Lesions and Reparative Process of Epidermis.” Minerva Ginecologica 62, no. 1: 1–5 (Beta‐glucano e terapia delle candidosi recidivanti e delle disepitelizzazioni cutanee iatrogene.). 20186110

[fsn370212-bib-0072] Pillai, R. , M. J. Redmond , and J. F. Röding . 2005. “Anti‐Wrinkle Therapy: Significant New Findings in the Non‐Invasive Cosmetic Treatment of Skin Wrinkles With Beta‐Glucan.” International Journal of Cosmetic Science 27, no. 5: 292. 10.1111/j.1463-1318.2005.00268_3.x.

[fsn370212-bib-0073] Pillemer, L. , and E. Ecker . 1941. “Anticomplementary Factor in Fresh Yeast.” Journal of Biological Chemistry 137, no. 1: 139–142. 10.1016/S0021-9258(18)72984-0.

[fsn370212-bib-0074] Piquero‐Casals, J. , D. Hexsel , J. F. Mir‐Bonafé , and E. Rozas‐Muñoz . 2019. “Topical Non‐Pharmacological Treatment for Facial Seborrheic Dermatitis.” Dermatology and Therapy 9: 469–477. 31396944 10.1007/s13555-019-00319-0 PMC6704200

[fsn370212-bib-0075] Qi, C. , Y. Cai , L. Gunn , et al. 2011. “Differential Pathways Regulating Innate and Adaptive Antitumor Immune Responses by Particulate and Soluble Yeast‐Derived β‐Glucans.” Blood 117, no. 25: 6825–6836. 10.1182/blood-2011-02-339812. 21531981 PMC3128477

[fsn370212-bib-0076] Quatresooz, P. , C. Piérard‐Franchimont , G. Szepetiuk , C. Devillers , and G. E. Piérard . 2009. “Fungal Chitin‐Glucan Scaffold for Managing Diabetic Xerosis of the Feet in Menopausal Women.” Expert Opinion on Pharmacotherapy 10, no. 14: 2221–2229. 10.1517/14656560903201699. 19743936

[fsn370212-bib-0077] Queiroz, L. S. , M. S. Nascimento , A. K. Cruz , et al. 2010. “Glucans From the Caripia Montagnei Mushroom Present Anti‐Inflammatory Activity.” International Immunopharmacology 10, no. 1: 34–42. 10.1016/j.intimp.2009.09.015. 19804847

[fsn370212-bib-0078] Ring, J. , A. Alomar , T. Bieber , et al. 2012. “Guidelines for Treatment of Atopic Eczema (Atopic Dermatitis) Part I.” Journal of the European Academy of Dermatology and Venereology 26, no. 8: 1045–1060. 10.1111/j.1468-3083.2012.04635.x. 22805051

[fsn370212-bib-0079] Riseh, R. S. , M. G. Vazvani , and J. F. Kennedy . 2023. “β‐Glucan‐Induced Disease Resistance in Plants: A Review.” International Journal of Biological Macromolecules 253, no. Pt 4: 127043. 10.1016/j.ijbiomac.2023.127043. 37742892

[fsn370212-bib-0080] Ross, G. D. , and V. Vĕtvicka . 1993. “CR3 (CD11b, CD18): A Phagocyte and NK Cell Membrane Receptor With Multiple Ligand Specificities and Functions.” Clinical and Experimental Immunology 92, no. 2: 181–184. 10.1111/j.1365-2249.1993.tb03377.x. 8485905 PMC1554824

[fsn370212-bib-0081] Ross, G. D. , V. Vetvicka , J. Yan , Y. Xia , and J. Větvičková . 1999. “Therapeutic Intervention With Complement and β‐Glucan in Cancer.” Immunopharmacology 42: 61–74. 10408367 10.1016/s0162-3109(99)00013-2

[fsn370212-bib-0082] Samuelsen, A. B. , A. Rieder , S. Grimmer , T. E. Michaelsen , and S. H. Knutsen . 2011. “Immunomodulatory Activity of Dietary Fiber: Arabinoxylan and Mixed‐Linked Beta‐Glucan Isolated From Barley Show Modest Activities In Vitro.” International Journal of Molecular Sciences 12, no. 1: 570–587. 10.3390/ijms12010570. 21340001 PMC3039967

[fsn370212-bib-0083] Schiano, I. , S. Raco , E. Cestone , M. Jesenak , Z. Rennerova , and J. Majtan . 2021. “Pleuran‐β‐Glucan From Oyster Culinary‐Medicinal Mushroom, Pleurotus Ostreatus (Agaricomycetes), Soothes and Improves Skin Parameters.” International Journal of Medicinal Mushrooms 23, no. 12: 75–83. 10.1615/IntJMedMushrooms.2021041519. 35381156

[fsn370212-bib-0084] Schreml, S. , R. M. Szeimies , L. Prantl , M. Landthaler , and P. Babilas . 2010. “Wound Healing in the 21st Century.” Journal of the American Academy of Dermatology 63, no. 5: 866–881. 20576319 10.1016/j.jaad.2009.10.048

[fsn370212-bib-0085] Son, H. J. , H. C. Bae , H. J. Kim , D. H. Lee , D. Han , and J.‐C. Park . 2005. “Effects of β‐Glucan on Proliferation and Migration of Fibroblasts.” Current Applied Physics 5, no. 5: 468–471. 10.1016/j.cap.2005.01.011.

[fsn370212-bib-0086] Sousa, P. , D. Tavares‐Valente , M. Amorim , J. Azevedo‐Silva , M. Pintado , and J. Fernandes . 2023. “β‐Glucan Extracts as High‐Value Multifunctional Ingredients for Skin Health: A Review.” Carbohydrate Polymers 322: 121329. 10.1016/j.carbpol.2023.121329. 37839841

[fsn370212-bib-0087] Sroka‐Tomaszewska, J. , and M. Trzeciak . 2021. “Molecular Mechanisms of Atopic Dermatitis Pathogenesis.” International Journal of Molecular Sciences 22: 4130. 33923629 10.3390/ijms22084130 PMC8074061

[fsn370212-bib-0088] Sun, T. , J. Li , Y. Qin , et al. 2020. “Rheological and Functional Properties of Oat β‐Glucan With Different Molecular Weight.” Journal of Molecular Structure 1209: 127944. 10.1016/j.molstruc.2020.127944.

[fsn370212-bib-0089] Sung, N.‐Y. , E.‐H. Byun , S.‐K. Kwon , et al. 2009. “Immune‐Enhancing Activities of Low Molecular Weight β‐Glucan Depolymerized by Gamma Irradiation.” Radiation Physics and Chemistry 78, no. 7‐8: 433–436. 10.1016/j.radphyschem.2009.03.022.

[fsn370212-bib-0090] Tejinder, S. , K. Bhupinder , and K. Harinder . 2000. “Flow Behavior and Functional Properties of Barley and Oat Water‐Soluble β‐D‐Glucan Rich Extractions.” International Journal of Food Properties 3: 259–274.

[fsn370212-bib-0091] Thieme, D. , G. Spilker , R. Lefering , and C. Weinand . 2016. “O2C Laser Doppler and Digital Photo Analysis for Treatment Evaluation of Beta‐Glucan Versus Provitamin Pantothenic Acid of Facial Burns.” Facial Plastic Surgery 32, no. 2: 225–231. 10.1055/s-0036-1579782. 27097145

[fsn370212-bib-0092] Tiwari, U. , and E. Cummins . 2009. “Factors Influencing β‐Glucan Levels and Molecular Weight in Cereal‐Based Products.” Cereal Chemistry 86: 290–301.

[fsn370212-bib-0093] Toklu, H. Z. , G. Sener , N. Jahovic , B. Uslu , S. Arbak , and B. C. Yeğen . 2006. “Beta‐Glucan Protects Against Burn‐Induced Oxidative Organ Damage in Rats.” International Immunopharmacology 6, no. 2: 156–169. 10.1016/j.intimp.2005.07.016. 16399620

[fsn370212-bib-0094] Tong, D. W. , and R. S. Barnetson . 1996. “Beta‐1,3‐D‐Glucan Gel in the Treatment of Solar Keratoses.” Australasian Journal of Dermatology 37, no. 3: 137–138. 10.1111/j.1440-0960.1996.tb01031.x. 8771866

[fsn370212-bib-0095] Torsekar, R. G. , and M. M. Gautam . 2017. “Topical Therapies in Psoriasis.” Indian Dermatology Online Journal 8, no. 4: 235–245. 10.4103/2229-5178.209622. 28761838 PMC5518573

[fsn370212-bib-0096] Vaithanomsat, P. , N. Boonlum , W. Chaiyana , and S. Tima . 2022. “Mushroom β‐Glucan Recovered From Antler‐Type Fruiting Body of Ganoderma Lucidum by Enzymatic Process and Its Potential Biological Activities for Cosmeceutical Applications.” Polymers 14, no. 19: 4202. 10.3390/polym14194202. 36236150 PMC9573635

[fsn370212-bib-0097] van den Berg, L. M. , E. M. Zijlstra‐Willems , C. D. Richters , M. M. Ulrich , and T. B. Geijtenbeek . 2014. “Dectin‐1 Activation Induces Proliferation and Migration of Human Keratinocytes Enhancing Wound Re‐Epithelialization.” Cellular Immunology 289, no. 1–2: 49–54. 10.1016/j.cellimm.2014.03.007. 24721111

[fsn370212-bib-0098] Vera, J. , R. Fenutría , O. Cañadas , et al. 2009. “The CD5 Ectodomain Interacts With Conserved Fungal Cell Wall Components and Protects From Zymosan‐Induced Septic Shock‐Like Syndrome.” Proceedings of the National Academy of Sciences of the United States of America 106, no. 5: 1506–1511. 10.1073/pnas.0805846106. 19141631 PMC2635803

[fsn370212-bib-0099] Wang, Q. , X. Sheng , A. Shi , et al. 2017. “β‐Glucans: Relationships Between Modification, Conformation and Functional Activities.” Molecules 22, no. 2: 257. 10.3390/molecules22020257. 28208790 PMC6155770

[fsn370212-bib-0100] Wang, S. , H. Zhou , T. Feng , et al. 2014. “β‐Glucan Attenuates Inflammatory Responses in Oxidized LDL‐Induced THP‐1 Cells via the p38 MAPK Pathway.” Nutrition, Metabolism, and Cardiovascular Diseases 24, no. 3: 248–255. 10.1016/j.numecd.2013.09.019. 24418375

[fsn370212-bib-0101] Wani, S. M. , A. Gani , S. A. Mir , F. A. Masoodi , and F. A. Khanday . 2021. “β‐Glucan: A Dual Regulator of Apoptosis and Cell Proliferation.” International Journal of Biological Macromolecules 182: 1229–1237. 10.1016/j.ijbiomac.2021.05.065. 33991557

[fsn370212-bib-0102] Witte, M. B. , and A. Barbul . 1997. “General Principles of Wound Healing.” Surgical Clinics of North America 77, no. 3: 509–528. 9194878 10.1016/s0039-6109(05)70566-1

[fsn370212-bib-0103] Wood, P. J. 2007. “Cereal β‐Glucans in Diet and Health.” Journal of Cereal Science 46: 230–238.

[fsn370212-bib-0104] Wu, L. , J. Zhao , X. Zhang , S. Liu , and C. Zhao . 2021. “Antitumor Effect of Soluble β‐Glucan as an Immune Stimulant.” International Journal of Biological Macromolecules 179: 116–124. 10.1016/j.ijbiomac.2021.02.207. 33667560

[fsn370212-bib-0105] Xiao‐xia, W. 2012. “Research Progress in Silkworm Protein Resources and Its Application in Food Industry.” Science and Technology of Food Industry.

[fsn370212-bib-0106] Xu, X. , P. Chen , L. Zhang , and H. Ashida . 2012. “Chain Structures of Glucans From Lentinus Edodes and Their Effects on NO Production From RAW 264.7 Macrophages.” Carbohydrate Polymers 87: 1855–1862.

[fsn370212-bib-0107] Yan, J. , W.‐D. Cai , C. Wang , et al. 2020. “Macromolecular Behavior, Structural Characteristics and Rheological Properties of Alkali‐Neutralization Curdlan at Different Concentrations.” Food Hydrocolloids 105: 105785. 10.1016/j.foodhyd.2020.105785.

[fsn370212-bib-0108] Yu, C. , H. Chen , D. Du , et al. 2021. “β‐Glucan From *Saccharomyces Cerevisiae* Alleviates Oxidative Stress in LPS‐Stimulated RAW264.7 Cells via Dectin‐1/Nrf2/HO‐1 Signaling Pathway.” Cell Stress & Chaperones 26, no. 4: 629–637. 10.1007/s12192-021-01205-5. 33880723 PMC8275741

[fsn370212-bib-0109] Yuan, H. , P. Lan , Y. He , C. Li , and X. Ma . 2019. “Effect of the Modifications on the Physicochemical and Biological Properties of β‐Glucan‐A Critical Review.” Molecules 25, no. 1: 57. 10.3390/molecules25010057. 31877995 PMC6983044

[fsn370212-bib-0110] Zhang, B. , W. Lan , and J. Xie . 2022a. “Chemical Modifications in the Structure of Marine Polysaccharide as Serviceable Food Processing and Preservation Assistant: A Review.” International Journal of Biological Macromolecules 223, no. Pt A: 1539–1555. 10.1016/j.ijbiomac.2022.11.034. 36370860

[fsn370212-bib-0111] Zhang, J. , X. Wang , V. Vikash , et al. 2016. “ROS and ROS‐Mediated Cellular Signaling.” Oxidative Medicine and Cellular Longevity 2016: 4350965. 26998193 10.1155/2016/4350965 PMC4779832

[fsn370212-bib-0112] Zhang, T. , Q. Guo , Y. Xin , and Y. Liu . 2022b. “Comprehensive Review in Moisture Retention Mechanism of Polysaccharides From Algae, Plants, Bacteria and Fungus.” Arabian Journal of Chemistry 15: 104163.

[fsn370212-bib-0113] Zhang, Z. , H. Chi , and R. A. Dalmo . 2019. “Trained Innate Immunity of Fish Is a Viable Approach in Larval Aquaculture.” Frontiers in Immunology 10: 42. 10.3389/fimmu.2019.00042. 30740103 PMC6355669

[fsn370212-bib-0114] Zheng, X. , F. Lu , X. Xu , and L. Zhang . 2017. “Extended Chain Conformation of β‐Glucan and Its Effect on Antitumor Activity.” Journal of Materials Chemistry B 5: 5623–5631. 10.1039/c7tb02649h 32264536

[fsn370212-bib-0115] Zhong, X. , G. Wang , F. Li , et al. 2023. “Immunomodulatory Effect and Biological Significance of β‐Glucans.” Pharmaceutics 15, no. 6: 1615. 10.3390/pharmaceutics15061615. 37376063 PMC10302218

[fsn370212-bib-0116] Zhu, F. , B. Du , and B. Xu . 2016. “A Critical Review on Production and Industrial Applications of Beta‐Glucans.” Food Hydrocolloids 52: 275–288.

[fsn370212-bib-0117] Zulli, F. , F. Suter , H. Biltz , and H. P. Nissen . 1998. “Improving Skin Function With CM‐Glucan, a Biological Response Modifier From Yeast.” International Journal of Cosmetic Science 20, no. 2: 79–86. 10.1046/j.1467-2494.1998.171740.x. 18505493

[fsn370212-bib-0118] Zykova, S. N. , K. A. Balandina , N. V. Vorokhobina , A. V. Kuznetsova , R. Engstad , and T. A. Zykova . 2014. “Macrophage Stimulating Agent Soluble Yeast β‐1,3/1,6‐Glucan as a Topical Treatment of Diabetic Foot and Leg Ulcers: A Randomized, Double Blind, Placebo‐Controlled Phase II Study.” J Diabetes Investig 5, no. 4: 392–399. 10.1111/jdi.12165. PMC4210076 25411598

